# Social media and intergenerational bonding through young adults’ communication with older family members

**DOI:** 10.3389/fsoc.2025.1643296

**Published:** 2025-12-01

**Authors:** Faycal Farhi, Riad Jeljeli, Samira Setoutah, Khaled Zamoum, Abdelouahab Boukhenoufa, Selami Saidani, Leila Feguiri

**Affiliations:** 1 Department of Communication and Media, Al Ain University, Al Ain, United Arab Emirates; 2 College of Arts, Sciences, Information Technology, and Communication, University of Kalba, Sharjah, United Arab Emirates; 3 Department of Communication, College of Arts, Sciences, Information Technology, and Communication, University of Al Dhaid, Sharjah, United Arab Emirates; 4 College of Communication, University of Sharjah, Sharjah, United Arab Emirates; 5 Department of Mass-Communication, Sultan Qaboos University, Seeb, Oman; 6 Department of Media and Communication, Mohamed Boudiaf University of M’Sila, M’Sila, Algeria

**Keywords:** social media, sustainability, intergenerational interactions, social convoy theory, United Arab Emirates (UAE)

## Abstract

Social media platforms have emerged as critical in changing family communication patterns. Particularly, they have widely transformed the family dynamics in the rich cultural dynamics regarding intergenerational bonding. Especially in countries like the United Arab Emirates, the role and impact of these platforms in promoting family relationships remain critical. This micro-level study explores how the UAE’s young generation uses social media platforms to connect with older family members and how these digital interactions strengthen families’ intergenerational solidarity, emotional resilience, and cultural continuity. Theoretically supported by social convoy theory, this research study used a qualitative design to highlight these digital exchanges’ patterns, communication styles, motivations, and perceived effects. The data collected from 15 young adults in Dubai, United Arab Emirates, indicated that social media played a critical role in maintaining family relationships across distances, particularly WhatsApp and Facebook. Participants use these tools to share daily updates, celebrate events, and express care, supporting emotional bonds host of young adults adapted their digital behaviours to include other relatives, guiding them in technology useful stop social media also supported preserving cultural practises through language, religious greetings common storytelling first of all participants highlighted this digital bonding, they acknowledge that face-to-face interactions lack emotions. Overall, these findings highlighted the potential of social media platforms to improve intergenerational communication, cultural continuity, and emotional resilience within diaspora families. The results further provided implications for family-centred sustainability practices, specifically in a multicultural society like the UAE, where conventional values and contemporary technologies continuously intersect.

## 1 Introduction

Social media platforms have revolutionized global communication, enabling individuals worldwide to connect instantly (Kahne and Bowyer, 2018). Since their emergence in the early 2000s, these platforms have become a refined digital space supporting different interaction forms, including text, video, and multimedia content (Yu and Abbas, 2022). The broader adoption of popular platforms, i.e., Facebook, Twitter, Instagram, and LinkedIn, has significantly impacted how people engage on personal, professional, and social levels. However, the role of social media extends far beyond just interpersonal communication. It has become deeply ingrained in almost every part of daily life, making it a central part of modern society. By 2023, more than 4.6 billion people worldwide were actively using social networking platforms, emphasizing the global scale of this trend (Gazi et al., 2023). These platforms serve multiple purposes: spreading information, promoting dialogue, providing entertainment, and allowing users to share their lives in a digitally connected world. The ability to form virtual links has greatly improved how people interact and communicate across geographical boundaries. Studies consistently show high levels of engagement with social media across virtually every country studied so far (Tadpatrikar et al., 2021). According to Al-Khalili and Subari (2023), one of the critical benefits of social media for families is its ability to help preserve close relationships despite physical distance. When family members live far apart, these platforms offer an important way to stay in touch, share updates, and experience significant moments together in real time. Beyond just communication, social media also provides a room for emotional support, allowing family members to provide encouragement and share helpful resources during challenging times.

In a country like the United Arab Emirates (UAE), social media and smartphones have greatly strengthened family relationships by helping loved ones stay interconnected, regardless of distance (Youssef et al., 2024). The current study also explores how these social media platforms affect and sustain Intergenerational bonding among families, providing strong venues for communication and interactions. As Gjylbegaj and Abdi (2019) also argued, these technologies act as important tools, helping families to preserve regular contact through video calls, messaging apps, and social media updates. Such digital communication permits the exchange of personal stories and provides emotional support, promoting a sense of proximity. These platforms especially open up prospects for intergenerational bonding and sharing online experiences. Whether participating in virtual communities, posting photos and videos, or attending digital events. These interactions help support bonds and create a feeling of harmony. Social media also allows families to collaborate on projects and participate in discussions that promote connection and even spark new digital rituals (Ali et al., 2025; Smith et al., 2021). Ahmed and Eltahir (2023) cited an example of these platforms for playing a key role in sharing important updates, including health and safety information during the COVID-19 pandemic in the United Arab Emirates (UAE). They also helped families uphold traditions and celebrate special moments together. From birthdays to holidays, virtual gatherings have ensured that important events are experienced and enjoyed, keeping the spirit of togetherness alive. As noted by Mahmoud and AhmedShafik (2020), social media and digital tools have reinforced family relationships by making it easier to communicate, share experiences, and support one another across distances. These platforms help strengthen emotional bonds, sustain traditions, and promote a sense of togetherness, even during physical detachment or family shifts like divorce. They are key in improving family relationships and personal wellbeing in the digital age.

Therefore, this study aims to explore the role and effect of social media on intergenerational bonding among families in the United Arab Emirates (UAE). However, the notable and distinctive aspect is exploring this role through the lens of communication, making social media a strong venue to ensure familial sustainability and resilience. Especially, in a traditional and culturally enriched society like the United Arab Emirates (UAE), the role of social media can be acknowledged based on influences like cultural transmission and intergenerational bonding. However, despite the importance of this topic, existing studies mainly focused on two areas. First, existing literature indicated an overindulgence in social media, leading to family detachment and isolation among young adults. Second, studies suggest that social media positively affected family bonding during the healthcare crisis, i.e., COVID-19. Consequently, this study focuses on the role of social media in reinforcing family bonding according to the young users. Also, this study fills the second gap as the current post-pandemic era indicates a sustained shift in technology-user relations, highlighting current research as providing insights into the technology shift as a critical phenomenon before and after the pandemic. The primary questions in this study involve:

*RQ1*: How does social media affect intergenerational bonding among young adults and older family members in the United Arab Emirates (UAE)?

## 2 Conceptualising social convoy in social media affecting family bonding

Research into social relations has been crucial in deepening our understanding of social and cultural processes of life and ageing. Social relations are widely seen as important to overall wellbeing and are known to have long-lasting and meaningful impacts on socio-cultural aspects of life (Antonucci et al., 2014). The social convoy model suggests that people are surrounded by a network of supportive individuals who stay with them through different phases of life. These relationships vary in how close they are, the kind of support they offer, emotional or practical, and how frequently and where people interact (Fuller et al., 2020). Personal factors like age and gender, as well as life circumstances, i.e., social roles and cultural values, shape these social networks’ size, makeup, and quality (Antonucci et al., 2014). Linking these networks to family and intergenerational bonding is essential in a person’s social, cultural, and psychological wellbeing. To evaluate them, individuals are asked to sort their most important relationships into three circles, each representing a different level of emotional closeness. For instance, this study proposed the role of social media platforms in sustaining intergenerational bonding among family members, where young individuals prefer staying connected with older members. In this context, **t**he convoy model of social relationships supports the idea that individuals live surrounded by emotionally close and meaningful people, such as family members. This group, or “convoy,” offers emotional support, practical help, and a sense of validation throughout different life stages (Growiec, 2023). Relevant to the current study, Field (2010) argued that social relations with family members further enable people to continue their goals by gathering resources through social networks, including digital platforms. In relation to the propositions of social convoy theory and social media in the current era (Ali et al., 2024), also witnessed this phenomenon where social media use is regulated by parents, leading to increased confidence and robust communication practices. Besides, Ali et al. (2024) found that social media use positively affects parent–child relationships and strengthens children’s exposure to the social world and secure online activities in Pakistan. Using social media for intergenerational bonding also indicates “Heterophilous Interactions,” where people from different age groups interact to maintain social ties (Kadushin, 2012). Despite these individuals differing in age, gender, and social and economic status, social media interactions are significant in sustaining intergenerational bonding in the best possible manner.

## 3 Research methodology

This research includes an exploratory qualitative design to acquire an in-depth understanding (Agius, 2013) of social media in sustaining the social and cultural integrity in the UAE, from the intergenerational family bonding perspective. The data were acquired from young adults residing in the UAE using face-to-face interviews. An interview questionnaire was designed, including four root questions. The questionnaire is designed and further assessed by two subject experts (one senior researcher from media and cultural studies, and one assistant from sociology). Both experts carefully evaluated the questionnaire and suggested some revisions to match the study objectives and problems, to ensure the validity of results. Table 1 presents the interview questionnaires used in the current study. The interviews are conducted online using Microsoft Teams from March 21st 2025, to May 3rd 2025. Each interview lasted 25–40 min. Once the data were gathered, the transcriptions were obtained and carefully scrutinized for the analysis.

**Table 1:** Interview questionnaire.

S/r. no	Questions
1.	How do you use social media to stay connected with your older family?
2.	In your opinion, what role does social media play in maintaining or improving your relationship with the older family members?
3.	In your opinion, how does social media affect cultural continuity by passing down traditions in your family?
4.	What challenges or benefits do you experience when communicating with older family members through social media?

### 3.1 Sampling procedures

The current study population involves young adults residing in the United Arab Emirates (UAE). However, a purposive sampling approach is employed to specify the sampling strategy further, and homogeneous and criterion sampling approaches are preferred. The participants are young adults aged 18–30 in Dubai, UAE. Dubai is selected due to its diverse population, strong digital infrastructure, and distinct blend of traditional and modern family values. The sample size is specified using the recommendation of Hu and Chang (2017), suggesting a sample size of 12–50 participants is sufficient for a qualitative study. Thus, a sample size of 15 participants was selected with special consideration for saturation (Fusch and Ness, 2015), where recruitment ceased once no new themes or insights emerged. Table 2 summarizes participants’ demographics, including their gender, age, occupation, and qualification. The participants’ details are hidden as primary research ethics (Dooly et al., 2017). Informed consent was also obtained before interviewing, and they were assured that the researchers would refrain from using their information commercially.

**Table 2:** Personal details of study participants.

Codes	Gender	Education	Age	Occupation
Participant 1	Female	Bachelors	25	PR professional
Participant 2	Male	Diploma/certification	29	Driving instructor
Participant 3	Female	Masters	24	Teacher
Participant 4	Male	Bachelors	26	Engineer
Participant 5	Female	Masters	27	Social media manager
Participant 6	Male	Masters	30	Administrative officer
Participant 7	Male	Bachelors	29	Sales manager
Participant 8	Female	Doctorate	29	University lecturer
Participant 9	Male	Undergraduate	18	Student
Participant 10	Male	Undergraduate	21	Students
Participant 11	Female	Masters	30	Business owner
Participant 12	Female	Masters	26	Teacher
Participant 13	Female	Masters	28	Research analyst
Participant 14	Female	Undergraduate	21	Student
Participant 15	Female	Undergraduate	19	Student

### 3.2 Data analysis approach

The data analysis is conducted using Interpretive Phenomenological Analysis (IPA). According to Smith and Nizza (2022), IPA is a widely preferred qualitative approach that provides understanding about how individuals make sense of their experiences. The relevant approach helps explain one’s own experience of a phenomenon in a distinct manner. Hence, IPA involves a systematic process that includes reading and re-reading data. Further, initial notes were taken that helped to develop emergent themes. The researchers later searched for relations across themes, which helps to look for patterns across cases. Finally, results were interpreted and reported.

## 4 Analysis and results

This section contains analysis and findings acquired from the gathered data. As Interpretive Phenomenological Analysis (IPA) is applied, data for each question is carefully analyzed. Notably, all the questions are represented along with the analysis-based tables to indicate the prevalent themes and dominant responses. Besides, existing literature is extensively discussed in relation to the current study. Each question contains a maximum of 2–3 themes that reflect the responses from the study participants.

### 4.1 Question 1: how do you use social media to stay connected with your older family?

Responses to these questions further generated three preceding themes: WhatsApp as the core connector, family bonding through media sharing and group chats, and digital role adaptation. Table 3 provides details of themes and key insights regarding social media use for staying connected with older family members.

**Table 3:** Details of themes and key insights regarding social media use for staying connected with older family members.

Key themes	Description	Key insights across participants
WhatsApp is the core connector	WhatsApp serves as the most accessible and widely used platform for intergenerational communication.	Participants mentioned frequent communication with older family members through WhatsApp. Simplicity and familiarity made it the preferred platform across generations.
Family bonding through media sharing and group chats	Group chats and sharing images, videos, and daily updates improve emotional connections.	An emphasis on the value of family group chats (on WhatsApp and Facebook), where they engage with elders by sharing pictures, greetings, and helping maintain a sense of togetherness.
Digital role adaptation	Young adults adapt their digital behaviour to stay consistent with the older relatives’ preferences and abilities.	Many avoid complex/youth-centric platforms for communication with elders. Some also took on a guiding role, helping grandparents handle social media tools, which reinforced bonding.

#### 4.1.1 Theme 1: WhatsApp is the core connector

A study by Taipale and Farinosi (2018) examined WhatsApp instant messaging within families in Finland and Italy, two countries representing distinct familial and communication traditions. It aimed to analyze how WhatsApp supported intergenerational communication in multigenerational family structures. Data collected through qualitative methods revealed that WhatsApp significantly improved family interactions across generations. Its effectiveness is derived from two main factors: the ability to communicate with all family members simultaneously, and its support for phatic communion through brief, casual messages. Besides, diverse message formats, i.e., text, voice, photos, and videos, allowed family members to adapt to one another’s communication choices and technological capabilities. Consistent with the relevant study, current results also indicated WhatsApp as the core platform preferred by study respondents. For example, participant 5 argued.

“Uhhh… I mostly talk to my grandma on WhatsApp. I can easily access her there as she stays online most of the time. We send each other good morning messages and sometimes notes. I also share videos and posts with her because she enjoys them all. She loves it when I send her pictures from my day.”

Consistent with participant 5, participant 2 opined that.

“I live away from home. I currently reside in Dubai, and my parents live in Sharjah. We use WhatsApp to do video calls, especially on weekends. My dad is not good with texting, but he enjoys short calls that talk about life and laugh. I also keep my mom updated through messages or brief calls to stay connected.”

As noted by Procentese et al. (2019), one of the key benefits of having a WhatsApp family group is the ease and proximity it brings to communication. It allows family members to share updates, personal situations, or coordinate virtual gatherings quickly. Group chats are more suitable than calling each person individually, particularly when everyone is busy. Getting immediate advice or information through chat is the perfect option. However, the true value of WhatsApp groups is in creating and using them thoughtfully, without overdoing it.

#### 4.1.2 Theme 2: family bonding through media sharing and group chats

The second theme generated from data indicated that they prefer family bonding through media sharing and group chats using WhatsApp. In this regard, participant 1 responded that.

“We have a big family group on WhatsApp. My cunts and uncles are added there. Me My cousins and I also stay connected. Everyone shares photos, jokes, and little updates. I believe that it is a small but nice online group because even my older aunts and uncles feel included, which is a good thing.”

da Conceicao (2022) also examined how WhatsApp family groups maintained communication bonds among families. The researcher applied new media theory and used a qualitative descriptive method, with data collected through interviews, observations, and document analysis. The results revealed that WhatsApp groups improved emotional bonds, supported daily communication, and worked as a platform for sharing information and entertainment within families.

“Whenever there is a birthday or celebration, I send pictures to the family group. Sharing small happiness and updates makes me feel linked to my family members. My grandparents always respond with emojis or little prayers. It makes them happy. If they are happy, I am happy.”

Previous research by Sukrillah and Ratnamulyani (2017) also explored communication patterns within the WhatsApp family groups. Their results indicated that the group setting gave rise to various distinct communication moments. These included expressions of friendship, playful teasing or mild bullying, displays of self-identity or presence, and moments of sarcasm or gentle reprimand, all happening naturally within the flow of family conversations in the WhatsApp group.

#### 4.1.3 Theme 3: digital role adaptation

According to Farhi et al. (2021), WhatsApp is a crucial social media platform that enables users to communicate through text and voice messages and share photos, videos, documents, and locations. Its myriad features make it an effective tool for personal and group communication. As a result, WhatsApp helps promote a culture of connectivity among tech-savvy generations and serves as a useful medium for maintaining relationships and accessing information. Thus, the third theme generated for the first question highlighted WhatsApp’s core importance and role as the most preferred platform to communicate with family. According to Participant 9,

“You know, some apps are trending; everyone uses them. My friends mostly use, My friends mostly use Instagram and Snapchat, and they keep posting stuff there. I do not prefer using both with my grandparents. They do not understand those apps. I just message them on WhatsApp because it is easier for them.”

In line with participant 9, participant 14 also indicated WhatsApp as the preferred platform, not only by participants but also by older family members. As stated.

“I try to teach my grandparents more features of WhatsApp. They are not fast learners, but they can try to use WhatsApp well. For example, I showed my grandma how to send voice notes last month. She was so excited. Now she sends me one every morning. Teaching her new features makes us even closer, and I am really happy.”

Consistent with the current results, Nouwens et al. (2017) also highlighted that WhatsApp, like other messaging apps, was used by individuals to create distinct communication spaces, including those for family interaction. It showed that users frequently chose WhatsApp for family communication due to its familiarity, ease of use, and emotional significance. Family groups on WhatsApp developed communication norms, purposes, and boundaries, which adapted over time based on relationship changes and technological factors.

### Question 2: in your opinion, what role does social media play in maintaining or improving your relationship with the older family members?

The data regarding the second study question further helped generate two major themes. These themes include bridging the distance and strengthening everyday bonds. Table 4 represents the details of themes and key insights regarding the role of social media in maintaining or improving relations with older family members.

**Table 4:** Details of themes and key insights regarding the role of social media in maintaining or improving relations with older family members.

Key themes	Description	Key insights across participants
Bridging the distance	Social media reduces emotional and physical distance between generations.	Participants noted that platforms like WhatsApp allow them to stay emotionally close despite living far apart.
Strengthening everyday bonds	Causal daily interactions through social media reinforce ongoing relationships.	Participants highlighted that sharing updates, photos, or greetings helps maintain a sense of belonging and routine.

#### 4.2.1 Theme 1: bridging the distance

The first theme from the data indicated a strong, constructive, and supportive role of social media platforms in sustaining communication among family members. Study respondents indicated an overall positive role of online platforms. However, a greater emphasis on Facebook and WhatsApp is seen. For instance, participant 1 responded.

“My grandmother lives in a different city and I do not visit her often, but WhatsApp helps us stay in touch. I send her voice notes every few days, and sometimes she replies with long audio messages or emojis. It makes me feel like I am still part of her daily life, even from far away.”

A study by Abel et al. (2021) explored how social media supported family relationships, identity, and collaboration among families with limited opportunities for face-to-face interaction. Data collected from a mixed-methods systematic review of empirical research published between 1997 and 2019 identified four main themes: how families operated in digital spaces, how they expressed identity through shared stories and rituals, the nature of their online communication, and issues related to privacy, conflict, and relationship quality. The study indicated the significance of family members who sustained social media relationships. Response by participant 15 also indicated the importance of social media and the role of active family members in sharing their routine life, leading to constant communication and strong family bonding. As opined.

“Before using social media, I only talked to my grandparents on special occasions or if there was a family event. But now, even if I am busy with university, I can send a message or a quick photo on Facebook Messenger, and they know I am thinking about them. It’s changed how we often communicate with our family members.”

As noted by Ali and Pasha (2024), one key advantage social media offers families is the ability to stay connected despite physical distance. These platforms create an important channel for constant interaction and real-time sharing of personal moments for those living apart. Besides, social media can serve as a space for emotional support, allowing family members to motivate one another and exchange helpful resources.

#### 4.2.2 Theme 2: strengthening everyday bonds

According to Khalili et al. (2024), also stated that social media has greatly changed how people link and communicate inside and outside their families. Platforms like Instagram, Facebook, Twitter, WhatsApp, and others now play a prominent role in everyday life, providing new and extended ways for people to stay in touch and interact. Accordingly, the second theme indicated the role of social media in strengthening everyday bonds between family members. For example, participant 3 revealed that.

“Sometimes I share random things in the family group, like a picture of my lunch or a funny meme, and my older relatives always respond. My aunt replies with laughing emojis, and my uncle usually sends a thumbs-up. It may seem small, but it makes me feel like we are still connected in our everyday lives.”

Consistent with the previous response, participant 8 also indicated an active role of social media in strengthening family bonds, as argued.

“My mom’s older sister always used to feel left out because she is not very tech-savvy, but since we started posting in the group regularly, she has been replying with simple messages. Just saying “thank you” or sending a photo of what she’s cooking brings us closer. It feels like she is a part of our daily routine now.”

Hence, social media allows families to engage in shared experiences and interact collectively (Kadushin, 2012). Families can maintain their sense of unity through online groups, multimedia sharing, and virtual gatherings. These venues also support joint activities and conversations that encourage stronger bonds and can lead to developing new digital practices (Nouwens et al., 2017).

### 4.3 Question 3: in your opinion, how does social media affect cultural continuity by passing down traditions in your family?

The third question further helped generate two central themes: Celebrating traditions online, language and expressions. Table 5 below provides details of themes and key insights regarding social media effects on cultural continuity.

**Table 5:** Details of themes and key insights regarding social media effects on cultural continuity.

Key themes	Description	Key insights across participants
Celebrating traditions online	Social platforms help keep cultural and religious practices visible and engaging for youth.	Young adults indicated that festivals, religious days, and traditional foods are often celebrated digitally with photos/videos.
Language and expressions	Sharing content in native languages or using traditional idioms helps preserve cultural identity.	Participants revealed that even simple chats or greetings in native languages help reinforce cultural relationships.

#### 4.3.1 Theme 2: celebrating traditions online

A study by Wibowo et al. (2023) aimed to explore how social media influences cultural interactions within society. Data gathered using a qualitative approach indicated that while social media significantly contributes to cultural exchange, it also improves the process of cultural integration. It introduced significant opportunities like an introduction to culture, acceptance, and bonding between diverse people. Thus, the necessity of using social media thoughtfully to promote deeper and more harmonious cultural integration in the digital age is emphasized. In line with the study by (Wibowo et al., 2023), study participants also highlighted the role of social media in facilitating the celebration of cultural traditions. According to participant 6,

Participants 10 and 11 also indicated that social media provides opportunities to celebrate traditions online. As opined.

“When there are special occasions such as Eid, we post photos of food, clothes, and prayers in their family groups, even if some of us are in different countries, it feels like we are celebrating together. It keeps our spirit alive and reminds the younger ones what our traditions look like. It also helps us feel we are still close despite our geographical differences.” (Participant 10)

“Last year, my cousins and I made a short video about our family rituals and posted it on our Facebook and Instagram. My younger siblings and cousins watched it over and over again. It is a good way to teach them our traditions in a format they understand and enjoy. Social media is important, especially when we have to connect with family. I rely on online platforms to stay updated on the current happenings in my family.” (Participant 11)

As noted by Wei (2024) new media platforms offer a modern approach to sharing traditional cultural bonds, making it more appealing to younger generations. Preserving and promoting cultural heritage by providing access to similar identities, new media impacts the spread of traditional cultural practices.

#### 4.3.2 Theme 3: language and expressions

The third theme generated from the data indicated the role of social media in promoting language and expressions. The participants revealed that these platforms help them learn and feel associated with their cultural heritage by enabling them to communicate in their native language. As noted by participant 13,

“Well, my dad always writes messages in her mother tongue, and now I have started doing the same in our family group. Even though I am more comfortable speaking English, using our mother language makes me feel connected to my roots. Let me tell you that I’m not fluent in my mother tongue, but I enjoy speaking it with my family members.”

Ugwu (2024) also noted that social media platforms are seen as valuable tools in supporting language preservation, offering new possibilities to strengthen efforts to maintain and revitalize languages. Accordingly, participant 7 also opined that.

“I believe that family groups on social media are the best way to stay connected to our mother language. For example, my grandma sends jokes or blessings in our traditional language. I have to ask my mum to translate, but I love it because it is our culture, and I want to learn and use those words too.”

A study by Minhas and Salawu (2024) also examined the role of social media in preserving and promoting indigenous languages through the platforms Facebook and X (formerly Twitter). The results showed that these platforms are important tools for language preservation, enabling cultural expression, identity formation, and engagement in the digital age. The study also considered the positive social effects, evolving norms, and consequential representations linked with online language use.

### 4.4 Question 4: what challenges or benefits do you experience when communicating with older family members through social media?

Finally, three themes are generated from the last questions. These themes involved bridging the generation gap, Tech literacy and communication, and Emotional connection vs. surface talk. Table 6 indicates the details of themes and key insights regarding challenges or benefits when communicating with older family members through social media.

**Table 6:** Details of themes and key insights regarding challenges or benefits when communicating with older family members through social media.

Key themes	Description	Key insights across participants
Bridging the generation gap.	Social media helps sustain communication and emotional proximity regardless of age or distance.	Participants noted that using platforms like WhatsApp strengthens family bonds by enabling frequent and informal chats with each other.
Tech literacy and communication	Older family members struggle with understanding digital cues or using features.	Study participants highlighted misunderstandings, misuse of emojis and features, and slow replies as common challenges.
Emotional connection vs. surface talk	Social media supports daily communication but lacks in-person interactions.	Study participants felt that while messages and calls help sustain connection, deeper emotional sharing and proximity often remain limited.

#### 4.4.1 Theme 1: bridging the generation gap

According to Mainsah et al. (2016), the emergence of major social media platforms marked a turning point in how people connect and communicate globally. These platforms have been critical in promoting cultural exchanges, supporting family connectivity, and nurturing virtual communities. Social media has significantly altered how individuals connect and interact with their family and friends by enabling real-time communication and easy information sharing. Consistent with the relevant argumentation, the first theme concerning last questions also indicated the role of major social media platforms, i.e., WhatsApp and Facebook, as important in supporting connectivity and communication with their family. As participant 8 argued that.

“Let me give you an example. My maternal grandparents live in Jordan, and I am here in Dubai. We do not get to meet often, but WhatsApp makes it feel like we are still close. They send me prayers, and I send them photos of my everyday life. It is not long chats, but those small things keep us connected. Sometimes, just a heart emoji from my grandparents makes my day.”

Participant 11 also opined that.

“We have made a family group on WhatsApp and Facebook Messenger where all generations are included. It is such a good way to stay in touch with each other. My grandfather does not talk much on video calls, but sends audio messages. I love hearing his voice; it makes me feel that I am around him and that we are connected to our family roots and culture, even though we are in different countries.”

However, a study by Downs (2019) indicated contradictory results as they examined intergenerational communication and perceptions between Baby Boomers and Millennials in the United States. The study aimed to identify the primary rationales behind the disconnect between the two generations and explore how each group believed they were viewed by the other. Data collected through an online survey distributed to faculty and staff at a private institution revealed that negative perceptions were not inherently held between the generations. Rather, individuals tended to assume they would be judged negatively by others and adjusted their behaviour accordingly.

#### 4.4.2 Theme 2: tech literacy and communication

The second theme also indicated the importance of tech literacy and communication for older individuals. Participants revealed concerns about misusing certain expressions and a lack of understanding. As participant 4 argued.

“Uhhhhhhhh… My father still types with one finger sometimes accidentally sends random stickers or forward weird videos, which is problematic post up once he replied “LOL” thinking it meant “lots of love”, and it was a bit awkward and funny. But honestly, I find it sweet too. You all laugh and help him learn, it is part of the fun”.

According to Hobbs (2016), tech literacy has evolved significantly. Initially centred around understanding and producing printed information, it has extended to include different modern forms of communication. With the rise of social media, media literacy now also involves engaging in group collaboration and contributing to collective dialogue through content creation. It encompasses a broad set of knowledge and skills that enable individuals to actively participate in today’s media-driven society by accessing, interpreting, evaluating, and generating messages across multiple formats and platforms, including personal messaging venues. In line with this argumentation, participant 12 indicated.

“Sometimes my grandmother thinks I have seen your messages and have not replied in 2 min. In other words, she thinks that I am ignoring her. She does not understand the “last seen” features and takes it personally. I have explained that I may be at work, busy, or outside, but it is hard for her to grasp the idea of being online but not instantly available.”

According to Polanco-Levicán and Salvo-Garrido (2022), social media platforms stand out from other internet-based tools because the public widely uses them, supports user-generated content, and promotes active participation rather than passive consumption. These platforms enable individuals to create and share information regardless of professional media training. This aspect becomes even more significant given that people of all ages now engage with social media regularly.

#### 4.4.3 Theme 3: emotional connection vs. surface talk

According to Knapp et al. (2014), one of the most compelling elements of digital communication is how it affects emotional closeness and the feeling of being socially connected. While these platforms allow people to stay in touch instantly, they usually fail to express actual emotions due to the absence of body language and facial expressions. Researchers hold differing opinions, as some believe that tools like emojis, animated images, and video chats help fill the emotional gap created by the lack of physical interaction. Others argue that these features still fail to convey tone, intent, and subtle emotional cues accurately.

Thus, data also indicated a lack of emotional connection and surface talk as prominent aspects of social media communication with family members. For example, participant 5 argued.

“We message every day, things like ‘good morning’ or ‘have you eaten?’, which is nice. But I do miss the deeper conversations we used to have in person. Social media keeps us in touch, but understanding their feelings is not always enough”.

Participant 13 further added that.

“My grandfather is old school; he finds it hard to express emotions on chat. Even when we talk on video, it is brief and polite. Staying in touch this way is helpful, but I do not know how he is doing inside.”

“I am grateful we have WhatsApp to stay in touch. But honestly, it does not feel the same as sitting with my aunt and listening to her stories. In chat, it is just quick messages like ‘take care’ or ‘miss you.’ There is no real emotion behind it. I miss the warmth of her expressions, the pauses in her voice. Online talks are convenient, but they are a bit hollow sometimes.”

In their study, Zulkifli (2025) explored how Generation Z experiences and maintains emotional connections in digital communication. A phenomenological approach was used, involving in-depth interviews and thematic analysis. The results indicated that digital platforms enabled new forms of emotional intimacy through features like asynchronous interaction and multimodal expression. However, participants also faced challenges, including the lack of non-verbal cues and frequent message misinterpretation, which sometimes impeded deeper emotional bonds.

## 5 Conclusion

The convoy model brought significant insights into understanding social relationships, particularly within families and close social circles. It perceives these relationships as complex and comprised of different parts, i.e., structure, type, and quality. While earlier research mainly focused on how many relations a person had, later studies also began to look at how meaningful and supportive those relationships were. In the context of family and social convoys, this means not only considering how many relatives or close contacts someone has, but also how strong, supportive, and positive those relationships are throughout life (Fuller et al., 2020).

The study results emphasized the role of platforms like WhatsApp and Facebook in sustaining familial ties, mainly when physical distance exists between family members. These digital tools facilitated the exchange of daily updates, celebrations, greetings, and multimedia, which promoted a sense of belonging and mutual care. As noted by Antonucci et al. (2017) Social interaction has significantly transformed with the rise of new technologies that offer diverse ways to stay connected. Over time, society has moved from traditional face-to-face meetings and handwritten letters to innovations like the telegraph and telephone. Today, communication has become even more personal and digital, with tools like mobile phones, video calling apps like Skype and FaceTime, and platforms like Facebook enabling instant and widespread connectivity. In doing so, the current study indicated that young adults maintained their bonds with older relatives. They adapted their digital behaviours, avoiding certain platforms and guiding elders in using technology to ensure inclusive and respectful communication.

Furthermore, this study found that social media supports the continuity of cultural practices. Through sharing photos, religious greetings, and traditional expressions in native languages, participants preserved their cultural identity and educated younger family members about traditions. These virtual exchanges served as modern methods of cultural storytelling and familial reinforcement. The argumentation by Antonucci and Fiori (2010) also supports the current study outcomes. As noted, while older adults may be slower to adopt new technologies, they are usually encouraged to do so because of their desire to stay connected with family members. They learn to use smartphones or social media to maintain communication with their children and grandchildren.

Overall, these results highlighted the positive role of social media on international solidarity, cultural continuity, and emotional resilience in diaspora communities. It highlights the importance of considering digital media not only as a tool for communication but as a meaningful space where identities, relationships, and traditions are nurtured across generations. Adetomi and Jinjiri (2024) also supported the current study findings, considering social media platforms contribute positively to individual experiences and the family unit due to the recent increase in social media use and its effects on family dynamics. However, study participants raised that social media lacks emotional bonding compared to face-to-face interactions, which may indicate a strong gap for future studies. As noted by Tettegah (2016) social media plays a key role in reinforcing family relationships by promoting genuine social interaction among individuals. On the other hand, relationships formed through social media cannot replace the deep emotional connections and bonds in authentic family relationships and face-to-face interactions. Altogether, findings suggest that when used intentionally, social media ensures cultural sustainability, supports family dynamics, and supports the intergenerational transmission of cultural values in multicultural societies.

### 5.1 Implications and recommendations

Based on the findings and in-depth insights, this study provides some practical implications and recommendations.

- Older adults usually face challenges when using contemporary technologies. To address this, community sentences, family support programmes, and libraries should offer training sessions focused on primary smartphone usage, messaging applications like WhatsApp, and social media platforms like Facebook. These initiatives can help bridge the generational digital divide and enable older family members to stay engaged in family conversations, daily life updates, and celebrations.
- Families should be encouraged to use social media for brief checks and as a platform for deeper engagement. This involves regularly sharing family news, celebrating birthdays or achievements together, posting old family photos, and expressing emotional support. These actions can help sustain our robust emotional bonding despite physical distances, especially for families from different countries or cities.
- Social media platforms provide a strong space for the continuation of cultural practices. Families can share religious greetings, native language phrases, traditional recipes, and cultural customs through audiovisual content and messages. Search interactions help preserve cultural identity and enable younger generations to develop a deeper understanding of their roots, particularly in multicultural or diaspora communities where cultural engagement is a concern.
- Tech companies and developers should consider improving or creating features that serve older users, including simple user interfaces, voice navigation, and language options. This can make applications more accessible and user-friendly for elderly family members. Platforms also include family modes or guided tutorials that make it easier for older adults to stay connected with younger relatives without the frustration of complex settings.
- While digital communication keeps people connected, especially over long distances, it often lacks the emotional depth and warmth of face-to-face interactions. Families should be encouraged to complement online communication with in-person visits, video calls, voice notes and possibly. A balanced approach promotes stronger emotional bonds and helps sustain relationships across generations.

### 5.2 Limitations and future studies

Despite this study filling important gaps in the current literature and providing strong insights, it has some limitations. First, this study was conducted in Dubai, United Arab Emirates, which restricts the generalizability of the results. Future studies can expand on this study and conduct more research on the relevant phenomena in different geographical regions to overcome this limitation. The second limitation is that it uses a single qualitative method. Future researchers can combine different methods and delimit this scope by employing, for example, mixed methods to gain deeper insights. Finally, the third limitation involves data only from young adults, which is the core focus of the sampling approach. Applying a diverse sampling unit can further overcome this limitation in the future investigations.

## Data Availability

The raw data supporting the conclusions of this article will be made available by the authors, without undue reservation.
